# Simu2VITA: A General Purpose Underwater Vehicle Simulator

**DOI:** 10.3390/s22093255

**Published:** 2022-04-24

**Authors:** Pedro Daniel de Cerqueira Gava, Cairo Lúcio Nascimento Júnior, Juan Ramón Belchior de França Silva, Geraldo José Adabo

**Affiliations:** Division of Electronic Engineering, Instituto Tecnológico de Aeronáutica, São José dos Campos 12228-900, SP, Brazil; cairo@ita.br (C.L.N.J.); juan@ita.br (J.R.B.d.F.S.); adabo@ita.br (G.J.A.)

**Keywords:** Underwater Unmanned Vehicle, simulation, mobile vehicle dynamics

## Abstract

This article presents an Unmanned Underwater Vehicle simulator named Simu2VITA, which was designed to be rapid to set up, easy to use, and simple to modify the vehicle’s parameters. Simulation of the vehicle dynamics is divided into three main Modules: the Actuator Module, the Allocation Module and the Dynamics Model. The Actuator Module is responsible for the simulation of actuators such as propellers and fins, the Allocation Module translates the action of the actuators into forces and torques acting on the vehicle and the Dynamics Module implements the dynamics equations of the vehicle. Simu2VITA implements the dynamics of the actuators and of the rigid body of the vehicle using the MATLAB/Simulink^®^ framework. To show the usefulness of the Simu2VITA simulator, simulation results are presented for an unmanned underwater vehicle navigating inside a fully flooded tunnel and then compared with sensor data collected when the real vehicle performed the same mission using the controllers designed employing the simulator.

## 1. Introduction

Working with mobile vehicles often proves to be time consuming and, adding to the natural complexity of the matter, typically, there is also the additional burden of using complicated simulators. Simulators are a necessity when dealing with mobile vehicles since they allow the design team to increase its knowledge about the vehicle’s behavior and to test different scenarios. Quality of simulation is a requisite that rapidly grows in importance as the cost of equipment increases and the environment becomes more hazardous to operate.

Our research project aims to design an Underwater Unmanned Vehicle (UUV) to be used for the inspection of adduction tunnels in hydroelectric power plants. Initially, a search was done for possible simulators for this scenario that would satisfy the following requisites:

- Overall design simple and easy to understand, implicitly implying low structure complexity to configure an experiment.
- Easy description and modification of the vehicle physical parameters, its actuators and its sensors.
- Rapid testing of the different types of speed and position controllers.
- Simple to add features on top of it such as vehicle autonomous behaviors.
- Dynamic model completeness.

Nowadays, popular consolidated robotics simulators such as Gazebo [1] offer great physics accuracy in simulation and in customization, but its learning curve is steep. The same happens with rich-feature simulators such as Webots [2]. The set up of these simulators was considered too complicated by our team since they require complex file-based descriptions of the vehicle. Our solution does not require any file edition and offers a user interface to enter the parameters describing the dynamic model of the vehicle.

In this paper, we introduce our simulator named **Simu2VITA**, since it was developed on top of MATLAB/Simulink^®^ (MATLAB^®^ and Simulink^®^ are registered trademarks of The MathWorks, Inc., Natick, MA, USA) to simulate underwater vehicles designed by our team at ITA (Instituto Tecnológico de Aeronáutica, São José dos Campos, SP, Brazil). We have chosen MATLAB/Simulink^®^ due to its popularity among engineers and for being the academic and industry standard for the simulation of mechanical and electrical systems. To use this simulator, one needs to define explicitly only the vehicle parameters. The Simu2VITA software can be found at this repository, which includes an example of a simulation session and an animation produced by it.

The remaining sections of this article are organized as follows:

- Section 2 discuss popular UUV simulators and their main characteristics.
- Section 3 presents our simulator Simu2VITA and the considerations taken in its implementation. In addition to the presentation of the internal design functioning of Simu2VITA, this section also provides an overview on the modeling of a rigid-body vehicle and its actuators.
- Section 4 presents the simulation results for a UUV navigating inside a fully flooded tunnel and a qualitative comparison of these results with sensor data collected when the real vehicle performed the same mission, showing that Simu2VITA can be used for fast concept validation.
- Section 5 highlights the main points of the article and presents some possible future improvements for this work.

## 2. Background

There are well established free/public domain and commercial simulators for UAVs and UGVs (Unmaned Aerial and Ground vehicles) such as Gazebo [1], CoppeliaSim (former V-REP) [3], MATLAB/Simulink^®^ (see UAV Toolbox and Vehicle Dynamics Blockset) and X-Plane. UAVs and UGVs travel through air, which is a very low-density fluid. Differently, USVs (Unmaned Surface Vehicles) and UUVs are partially or fully submersed in water, which is a much denser and (for our purposes) incompressible fluid. Consequently, for the simulation of these vehicles, one has to consider hydrodynamics forces (namely buoyancy, drag and lift) and fluid inertia. Fluid inertia is modeled using the concept of *Added Mass* [4], which can be defined as the fluid mass deflected by the movement of the vehicle. The capacity of entering information about the Added Mass, and how much effort this simulation configuration step takes, should be a factor to consider when choosing a UUV simulator.

Designers of UUV simulators have basically two options: to add a fluid simulation extension to an existing UAV/UGV simulator (such as Gazebo or CoppeliaSim) or to design the simulator from the ground up. We show below that both options have been tried by researchers.

Gazebo from Open Source Robotics Foundation is a free open-source general-purpose 3D simulator that can handle multiple robots and has an extensive library of ready-to-use vehicle models. It was originally built to satisfy the need for a high-fidelity vehicle simulator in outdoor environments. Being in development since the early 2000s, the simulator now includes many sophisticated features such as over-the-network and cloud simulation. A simulated scenario configuration in Gazebo is done using SDF (Simulation Description Format) files [5] to describe the vehicle (in terms of its joints) and its environment. SDF is a XML-based format that was derived from URDF (Unified Robotic Description Format) [6].

However, Gazebo was not originally designed to simulate rigid bodies moving through a dense fluid such as water. Taking advantage of the Gazebo plugin architecture, an extension to add fluid simulation named *Fluids* [7] was created. However, its web page states that this plugin is experimental and outdated by now. There are also the *Buoyancy* [8] and *Lift-Drag* plugins [9], which allow simplified underwater vehicle simulations but have complicated parameter configurations such as the slope of the lift curve. The *Orca3* software package [10] offers a set of four Gazebo plugins (Thruster, Barometer, Buoyancy and Drag) but is specific for the BlueRobotics BlueROV2 UUV. The added mass effect on the dynamics of the UUV rigid body is not addressed by Gazebo on its own nor by any of its above-mentioned plugins.

A low-complexity alternative that also extends Gazebo is to use the free open-source UUV Simulator package [11]. It defines several plugins to implement hydrostatic and hydrodynamic effects, thrusters and sensors. This simulator allows the addition of the added mass information, which enhances the simulation accuracy. It also uses the modular design of Gazebo to enable the simulation of multiple underwater vehicles.

CoppeliaSim (formerly known as V-REP) from Coppelia Robotics is an open-source commercial (but free if used for educational purposes) simulator that competes directly with Gazebo. Both are stand-alone ground and aerial robot simulators with similar features such as over-the-network simulation (i.e., the robot and its controllers can be implemented in different computers) and capability extension using plugins. Both simulators also offer a few options for selection of the physics engine (https://gazebosim.org/blog/four_physics, https://www.coppeliarobotics.com/helpFiles/en/dynamicsModule.htm (accessed on 12 April 2022)), depending on the complexity of the particular case, for instance, if accurate 3D collision detection is important or robots with articulated links are used.

One alternative for CoppeliaSim to simulate UUVs is to select the Vortex Studio physics engine [12] (p. 56), which is a closed source and commercial engine but free for registered academic users. This engine implements the buoyancy, lift and drag forces and the added mass effect. A second alternative is to directly modify the selected CoppeliaSim physics engine to include fluid simulation and the added mass effect. Lu and Liu [13] have claimed they did that, but no further details were given.

The Webots Robot Simulator [2] from Cyberbotics Ltd. is also free open-source stand-alone simulator. It also shares many similarities with Gazebo and CoppeliaSim such as multiple robot simulation, collision detection between bodies, headless simulation over network (when the visualization is not required or is shown in a different machine and only the background computation of the simulation is performed in the simulator host machine), and ready-to-use models of sensors and robots. Webots also allows the addition of external forces to be added to the physics engine to create, for instance, a constant wind force affecting the vehicle. External communication with the simulator is possible using different approaches such as through a generic TCP/IP socket or using an API (Application Programming Interface) to an external application such as a program written in C/C++, Java, Python or MATLAB^®^. Webots is in many ways more suited for underwater simulation than Gazebo without plugins, since it includes fluid simulation (https://cyberbotics.com/doc/reference/fluid (accessed on 12 April 2022)) by design. However, apparently Webots does not consider the added mass effect since its documentation does not mention it.

Both Simu2VITA and the Gazebo-based UUV simulator use the same equations to simulate an underwater vehicle. Simu2VITA also uses a similar approach by building the simulation block on top of a more complete framework, in our case Simulink^®^. However, differently from all the other UUV simulators described above, Simu2VITA does not require the edition of a textual configuration to describe the vehicle. In Simu2VITA, all the pertinent information about the simulation is entered using its input menu interface (see Appendix A). When comparing CoppeliaSim with the Vortex Engine, Simu2VITA has the advantage of being an open-source software and is much easier to set up. In comparison to Webots, Simu2VITA can also accept inputs from outside the MATLAB/Simulink^®^ framework using functionalities from MATLAB toolboxes such as the Instrument Control Toolbox or the Robotics System Toolbox. However, unlike Gazebo, CopelliaSim and Webots, MATLAB/Simulink^®^ is a commercial software. Furthermore, Simu2VITA by itself does not include 3D visualization and uses the Simulink 3D Animation toolbox from MATLAB^®^ for this purpose.

For someone used to MATLAB/Simulink^®^, the learning curve to use Simu2VITA is very small. Its simplicity to achieve good quality rigid body simulation and its inherited communication and 3D visualization functionalities from the MATLAB/Simulink^®^ framework qualify it as a good choice for a UUV simulator and its use for rapid controller design and testing.

For a review of physics simulators for robotic applications in ground, medical, maritime and aerial environments, see Collins et al. [14].

## 3. The Simu2VITA Simulator

The Simu2VITA simulator implements the mathematical structure describing the laws of motion of an underwater vehicle. Such a structure is composed of the actuator module, allocation module and the dynamics module of the vehicle.

It is worthwhile noting that our solution can be easily adapted to simulate other types of vehicles (e.g., ground and aerial vehicles) by changing the values of the dynamic model which is described ahead, but this possibility will not be explored in this article. However, this article explains how to adapt Simu2VITA to simulate different types of underwater vehicles. Detailed instructions about how to configure our software can be found in Appendix A.

### 3.1. Simulator Description

Simu2VITA has three main modules describing different components of the vehicle. This modules are briefly described below, and more details are given in the subsections ahead.

- The **Actuator Module** implements the dynamic model of the actuators using for each of them an input signal saturation followed by a simple first-order system. Actuator inputs are handled by this module.
- The **Allocation Module** describes how the forces generated by the vehicle actuators are mapped into forces and torques acting on the body of the vehicle.
- The **Dynamics Module** has two main software components: the **kinematics component** that treats only geometrical aspects of the vehicle motion, and the **kinetics component**, which deals with the effect of forces and torques applied to the body of the vehicle.

**Figure 1:**
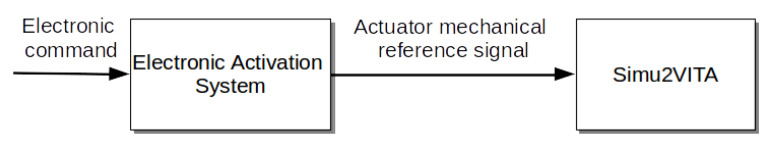
A simple schematic showing the logic to add some electronic activation dynamics of the actuators when using Simu2VITA.

On Simu2VITA, modeling is restricted to mechanical forces and torques acting in the vehicle and generated by its actuators. Therefore, an eventual electronic activation system of an actuator would have to be attached externally to the simulation block, as shown in Figure 1. A typical case is the translation of a PWM input signal to the expected thrust input signal of a propeller.

At this point, it is necessary to define the notation regarding vectors, matrices and linear transformations used herein. A vector v that is from some frame {U} is shortly written as $v_{U}$ or vUU, and if the same vector has to be transformed yet to another frame {W}, then it is denoted vWU. A matrix *P* that represents a linear transformation from frame {U} into frame {W} is written as PWU. Therefore, the expression linking $v_{U}$ and vWU is

(1) vWU=PWUvU,

and the transformation in the opposite direction is given by:

(2) PUW=PWU−1,

(3) vU=vUU=PUWvWU.

**Figure 2:**
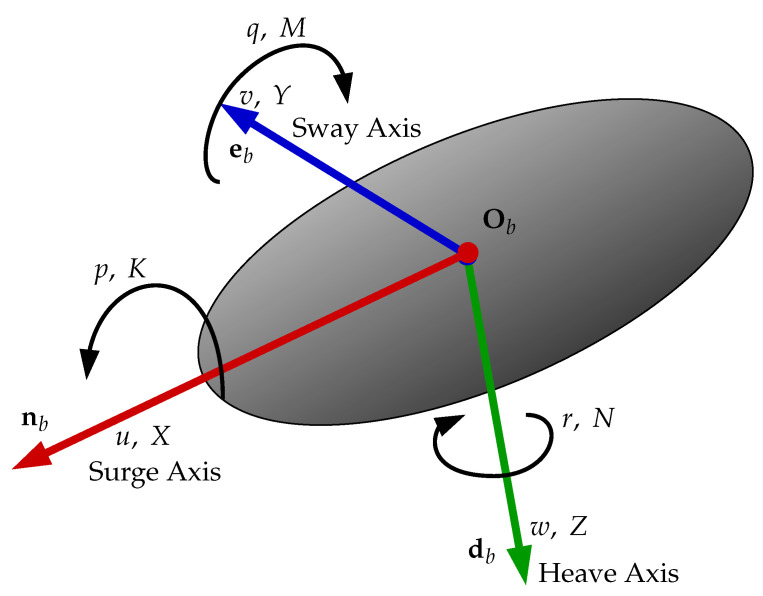
Definition of the body frame *b* of the vehicle. Note the components of $v_{b}$ and $\tau_{b}$ in each corresponding axis.

Figure 2 shows a three-dimensional frame attached to the body of some vehicle and the components regarding each axis of the body frame {b}. Observe that the frame {b} is defined according to the North–East–Down convention and centered at a chosen point $O_{b}$ in the body called the body frame origin. The independent vectors forming frame {b} are denominated:

- n for the forward pointing axis in red;
- e for the axis normal to the sagital plane of the vehicle in blue;
- and d for the axis pointing down in green.

Each axis of the body frame {b} is named according to the nomenclature defined by the Society of Naval Architects and Marine Engineers (SNAME) [15]. The vector n is named the Surge Axis, e is the Sway Axis and d is the Heave Axis. The vector $\upsilon_{b}={\left[u\;v\;w\right]}^{T}$ represents the linear velocity of the vehicle written in respect to its own body frame {b} and the components in each axis following the *Surge*, *Sway*, *Heave* order. The angular velocity is $\omega_{b}={\left[p\;q\;r\right]}^{T}$, with each component being the gyros around each axis. Both vectors can be put together in vector $v_{b}={\left[\upsilon_{b}^{T}\;\omega_{b}^{T}\right]}^{T}$. The forces and torques working on the vehicle body are all put in one single vector $\tau_{b}={\left[X\;Y\;Z\;K\;M\;N\right]}^{T}$, with *X*, *Y* and *Z* being the force components, and *K*, *M* and *N* being the torque components.

Next, the implementation of the three modules forming Simu2VITA are presented from a rear-to-front perspective. The Dynamics Module is presented in Section 3.1.1 and Section 3.1.2. Section 3.1.3 explains how the Allocation Module transforms the forces generated by the actuators to forces and torques acting on the vehicle. Finally, we show how an actuator is modeled and how the forces they generate are obtained in Section 3.1.4—the Actuator Module.

#### 3.1.1. The Dynamics Module-Kinematics Component

Defining the global reference frame adopted by the simulator as the NED (North–East–Down) frame convention and calling it {w}, the simulated vehicle state is described as follows:

1 The pose of the vehicle written with respect to (w.r.t.) the {w} frame,
  (4) ηwb=[pwbqwb]T,
  where pwb is the position and qwb is a unit quaternion [16] describing the orientation of the vehicle with respect to {w}. In addition, pwb=[ned]T, where *n*, *e* and *d* are the three Euclidean components in the {w} frame. The quaternion qwb=[q0ϵ]T has its real part as its first component and the imaginary part encapsulated in ϵ. Notice that quaternion vector qwb can be interpreted as “orientation of frame {b} in respect to frame {w}”.
2 The linear and angular velocities w.r.t. the vehicle’s own body frame
  (5) $$\begin{array}{c} v_{b}={\left[\upsilon_{b}^{T}\;\omega_{b}^{T}\right]}^{T}\;. \end{array}$$

The displacement of the vehicle w.r.t. {w} is calculated using η˙wb obtained from [17]

(6) η˙wb(vb,ηwb)=p˙wbq˙wbT=qwbυbqwb*Tq(qwb)ωbT,

with qwb* being the inverse of qwb [16] and $T_{q}\left(q\right)$ being a matrix with the form [17]

(7) $$\begin{array}{c} T_{q}\left(q\right)=\frac{1}{2}\left[\begin{array}{c} -\epsilon^{T}\\ q_{0}I_{3\times 3}+S\left(\epsilon \right) \end{array}\right]\;, \end{array}$$

where S(·) is the skew-symmetric matrix operator.

#### 3.1.2. The Dynamics Module-Kinetics Component

The differential equation describing the behavior of the vehicle [17] is

(8) Mv˙b+Mavbr+(C(vbr)+Ca(vbr))vbr+D(vbr)vbr+g(ηwb)=τb,

already accounting for hydrodynamics and hydrostatic components, where

- ${\dot{v}}_{b}$ is the acceleration vector of the vehicle.
- vbr is the relative velocity of the vehicle when accounting for constant water currents vbc,
  (9) vbr=vb−vbc,
  with
  (10) vbc=ucvcwc000T,
  where $u_{c}$, $v_{c}$, $w_{c}$ are, respectively, the components of the water current velocity in Surge, Sway and Heave.
- Matrix *M* is the rigid body Inertia Matrix and can be derived using Newton–Euler equations of motion. Here, *M* is defined using an arbitrary point $O_{b}$ in the body of the vehicle as the origin for frame {b} and has the structure
  (11) M=mI3×3−mS(rb)mS(rb)Ib.
  Vector rb describes the displacement of the center of gravity of the vehicle w.r.t. {b}, and it shall be informed when using the simulator. The scalar *m* is the mass of the vehicle. Matrix $I_{b}\in R^{3\times 3}$ is the Inertia Matrix defined around the origin of {b}. One possibility to obtain the value of $I_{b}$ is to first obtain the Inertia Matrix $I_{g}$ around $r_{b}$ and perform
  (12) Ib=Ig−mS2(rb).
- *C* is the Coriolis–Centripetal Matrix, and the form used here can be found using Newton–Euler method,
  (13) C=mS(ωb)−mS(ωb)S(rb)mS(ωb)S(rb)−S(Ibωb).
- $M_{a}$ is the Added-Mass Matrix, which accounts for the extra inertia added to the system because of the water volume the accelerating vehicle must displace in order to move through it. The information of the shape of the vehicle is embedded in this matrix [4]. This matrix is normally computed using an auxiliary numeric modeling software [18].
- $C_{a}$ is the Hydrodynamic Coriolis–Centripetal Matrix and has the following form
  (14) $$\begin{array}{c} C_{a}=\left[\begin{array}{cc} 0 & S(M_{a,11}\upsilon_{b}+M_{a,12}\omega_{b})\\ S(M_{a,11}\upsilon_{b}+M_{a,12}\omega_{b}) & S(M_{a,21}\upsilon_{b}+M_{a,22}\omega_{b}) \end{array}\right]\;. \end{array}$$
- *D* is the Hydrodynamic Damping Matrix, which is simplified in our model. Here, we assume the vehicle to perform relatively decoupled movements in each direction resulting in diagonal matrices for the linear and non-linear diagonal dumping.
- Vector g(ηwb) accounts for the static and hydrostatic forces acting on fully submerged vehicles, meaning gravitational force fwW=[00W]T and buoyancy force fwB=−[00B]T, with W=mg and B=ρg∇. Scalar *g* is gravity acceleration, ρ is the water density and ∇ is the volume displaced by the vehicle. Finally,
  (15) fbW=qwb−1fwW(qwb−1)*,
  (16) fbB=qwb−1fwB(qwb−1)*,
  (17) g(ηwb)=−fbW+fbBrb×fbW+bb×fbB,
  and observe that $b_{b}$ is the center of buoyancy in the body of the vehicle.
- $\tau_{b}$ is the vector of disturbing forces and torques applied to the vehicle in each axis of the body frame, including those generated by the actuators. We divide this vector into two main components as described in Equation (18)
  (18) τb=XYZKMNT=τba+τbe,
  where *X*, *Y* and *Z* are forces applied into the Surge, Sway and Heave Axis, respectively. Torques are *K*, *M* and *N* following roll, pitch and yaw movements, respectively. See Figure 2, with τbe encapsulating any external forces and torques from any source and τba coming from the actuators.

Internally, we compute the acceleration of the vehicle by simply isolating $\dot{v}$ in Equation (8) and transforming it into

(19) v˙b=(M+Ma)−1(τb+Mav˙bc−C(vb)vb−Ca(vbb)vbr−D(vbr)vbr−g(ηwb)),

considering v˙bc to be

**Figure 3:**
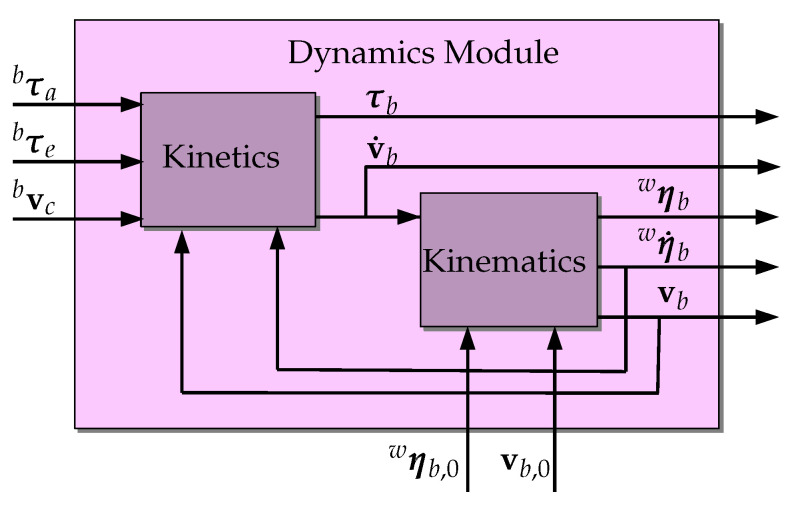
Logic representation of both Kinetics and Kinematics inside the Dynamics Model. Inputs and outputs are also represented.

(20) v˙bc=S(ωb)03×303×303×3vbc,

implicitly assuming the water current to be constant and irrotational [17]. This assumption is based on the premise that the UUV is not in an environment with currents that would generate perturbations in its torque state components, thus not leading it to rotate. This would be different if the vehicle was considered to be in an environment in the presence of waves. Figure 3 shows the internal flow of information, input, and output of this module. Observe that here, we also present the initial state vectors ηwb,0 and vbb,0 as inputs to the Kinematics part.

There are some methods to individuate the parameters here described such as the one presented by Wehbe and Krell [19] that uses support vector regression or the one from Karras et al. [20] that uses global derivative-free optimization.

#### 3.1.3. The Allocation Module

**Figure 4:**
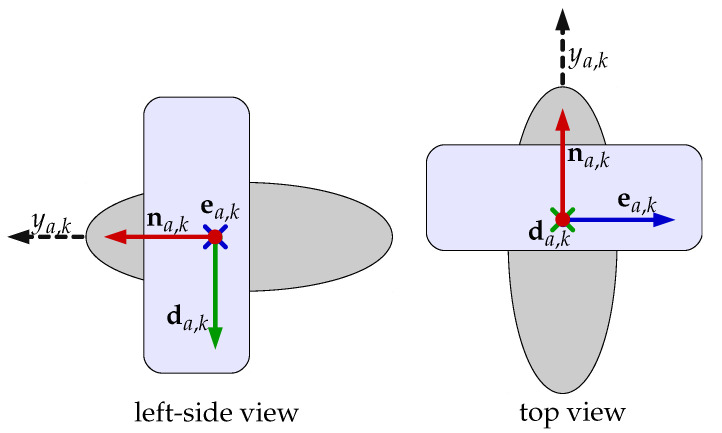
This image shows the direction of the force generated by a propeller. The left view shows a sideways view from the left of the propeller, the right view gives the top view. Observe that the output force vector $y_{a,k}$ is always aligned with the $n_{a,k}$ axis.

The Allocation Module task is to transform the output of the modeled actuators y into forces and torques inputs of the vehicle described by τba, i.e., a function $f\;:\;R^{n}\to R^{6}$ with n≥0 being the number of actuators contributing to the generation of forces and torques in all six degrees of freedom. Commonly, this transformation is linear, and so it is in our design. This linear transformation is firstly considered static, and later, a time-variant possible solution is shown. Equation (21) shows the static case transformation,

(21) τba=[XbaYbaZbaKbaMbaNba]T=Hya,

with $y_{a}$ being the vector containing the output of the actuators written in respect to these and matrix *H* being the allocation matrix, accounting for the contribution of each actuator in forces and torques acting in each axis of the vehicle. The computation of this matrix can be made pragmatically for the case where the actuators are propellers attached to the body of the vehicle. First, we consider the position of these actuators with respect to the {b} frame and their orientations using Euler angles. We denote the position of the *k*-th propeller in this case as pba,k=[nba,keba,kdba,k]T and its orientation as αba,k=[ϕba,kθba,kψba,k]T representing roll, pitch and yaw components. Now, assuming the propeller pushes the vehicle only in its na,k axis direction as in Figure 4, we compute nba,k as the resultant first column vector from the rotation matrix Rba,k describing the misalignment of the actuator frame with respect to the body frame of the vehicle

(22) Rba,k=[nba,keba,kdba,k]=R(ϕba,k)R(θba,k)R(ψba,k).

We then change the name of vector nba,k to express the distribution of the force $y_{a,k}$ generated by the *k*-th propeller in each axis of {b}.

(23) fba,k=[fbX,kfbY,kfbZ,k]T=nba,kT.

This way, the resultant force of the *k*-th propeller in each axis is given by

(24) yba,k=Xba,kYba,kZba,k=fba,kya,k.

The torque generated by the *k*-th propeller in the body is calculated using the cross-product of pba,k by $y_{a,k}$ resulting in

(25) Mba,k=Kba,kMba,kNba,k=[mbK,kmbM,kmbN,k]T︸mba,kya,k=pba,k×fba,kya,k.

**Figure 5:**
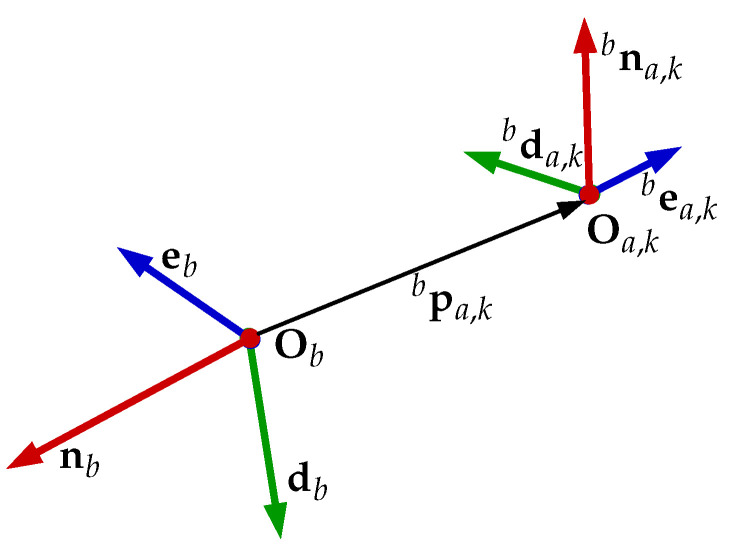
The representation of the frame of any *k*-th actuator with respect to the body frame {b} of the vehicle.

Figure 5 shows the geometric relation of pba,k and nba,k. It is now clear that the full allocation vector is hba,k=[fba,kTmba,kT]T, and we can align all allocation vectors in the matrix

**Figure 6:**
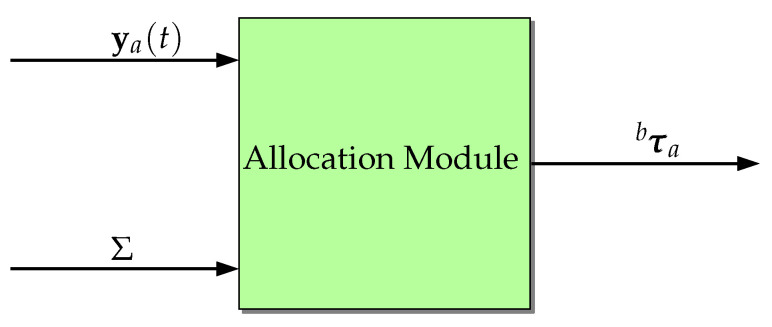
A graphic representation of the operation performed by the Allocation Module, with $y_{a}$ and *H* as inputs and τba as output.

(26) H6×n=[hba,1hba,2hba,3...hba,n]

with *n* being the total number of propellers, we obtain the allocation matrix. Now, multiplying *H* by a column vector y containing the forces coming from the propellers, the resultant forces and torques vector τba is generated and shown in Equation (21). Figure 6 shows a block diagram of this transformation.

**Figure 7:**
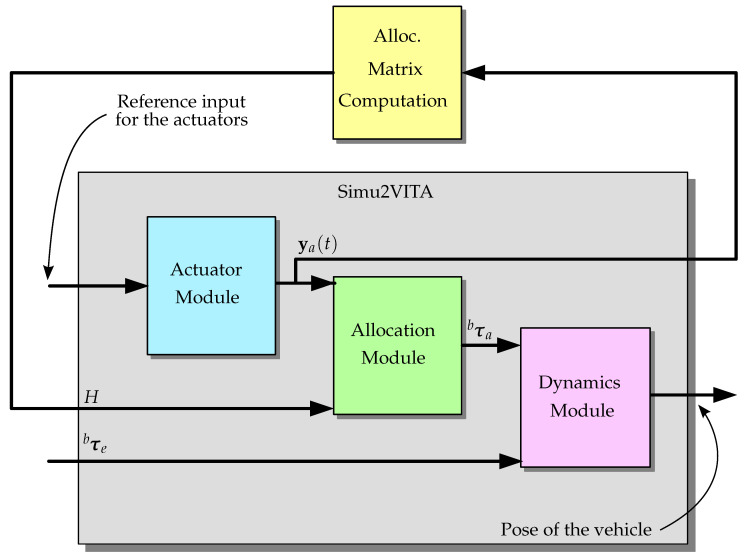
The logic representation of an external calculator for the allocation matrix in case of a moving propeller.

Observe that *H* can be time-dependent if the vehicle has movable actuators, for instance, a rotating propeller or even a fin for roll and pitch maneuvers. These rotating and movable actuators can be also modeled in the actuator module as will be shown in Section 3.1.4, but *H* will need to be calculated outside the simulator and this output fed back into Simu2VITA. For the simple case of a rotating propeller, the procedure we presented is the basis, with just the constant changing orientation needing to be tracked; see Figure 7. For fins, perhaps a non-linear approach is needed, and the final τba must be fed back directly using the τbe input of the Dynamics Module, as the Allocation Module internal machinery expects a matrix to perform a linear transformation, in this case H=0, i.e., the Allocation Module is bypassed. A future refining is to turn without the need for this bypass for the non-linear case of force allocation.

#### 3.1.4. The Actuator Module

The input of Simu2VITA represents the reference signal the actuators of the vehicle should follow. For instance, if the actuator is a propeller, the input reference signal should be the desired force to be generated by the actuator. In the case of a fin, the reference signal should be the desired fin angle. These input signals are handled by the Actuator Module. Each actuator is modeled as a saturation function followed by a first-order linear system with a user-defined time constant *T* (transfer function G(s)=1/(Ts+1)). Therefore, each actuator output $y_{a}\left(t\right)$ can be computed in time in closed form by

(27) $$y_{a}\left(t\right)=\exp\left[-\frac{\left(t-t_{0}\right)}{T}\right]y_{a}\left(t_{0}\right)+\frac{1}{T}\int_{t_{0}}^{t}\exp\left[-\frac{\left(t-\tau \right)}{T}\right]{\bar{u}}_{a}\left(\tau \right)d\tau \;,$$

where $y_{a}\left(t\right)$ is the output at time *t*, $t_{0}$ is the initial simulation time, $y_{a}\left(t_{0}\right)$ is the initial state, and ${\bar{u}}_{a}\left(t\right)$ is the limited input signal received by the actuator. Therefore, ${\bar{u}}_{a}\left(t\right)$ is defined as

(28) $$\begin{array}{c} {\bar{u}}_{a}\left(t\right)=sat\left(u_{a}\left(t\right),u_{min},u_{max}\right)=\left\{\begin{array}{cc} u_{min} & \mathrm{if}\;u_{a}\left(t\right)<u_{min}\;,\\ u_{a}\left(t\right) & \mathrm{if}\;u_{min}\leq u_{a}\left(t\right)\leq u_{max}\;,\\ u_{max} & \mathrm{if}\;u_{max}<u_{a}\left(t\right)\;, \end{array}\right. \end{array}$$

with $u_{min}$ and $u_{max}$ being, respectively, the lower and upper limit values for the actuator input signal $u_{a}\left(t\right)$. Note that the actuator output $y_{a}\left(t\right)$ is also bounded by $u_{min}$ and $u_{max}$.

Since a vehicle usually has multiple actuators, we need to define some useful vectors to express the whole system in a compact form using

(29) $$\begin{array}{ccc} y_{a}\left(t_{0}\right)\; & = & \;{\left[y_{a,1}\left(t_{0}\right)\cdots \;y_{a,n}\left(t_{0}\right)\right]}^{T}\;, \end{array}$$

(30) $$\begin{array}{ccc} T\; & = & \;{\left[T_{1}\cdots \;T_{n}\right]}^{T}\;, \end{array}$$

(31) $$\begin{array}{ccc} u_{a}\left(t\right)\; & = & \;{\left[u_{a,1}\left(t\right)\;\cdots \;u_{a,n}\left(t\right)\right]}^{T}\;, \end{array}$$

(32) $$\begin{array}{ccc} u_{\min}\; & = & \;{\left[u_{min,1}\cdots \;u_{min,n}\right]}^{T}\;, \end{array}$$

(33) $$\begin{array}{ccc} u_{\max}\; & = & \;{\left[u_{max,1}\cdots \;u_{max,n}\right]}^{T}\;, \end{array}$$

(34) $$\begin{array}{ccc} {\bar{u}}_{a}\left(t\right)\; & = & \;sat(u_{a}\left(t\right),u_{\min},u_{\max})\;, \end{array}$$

where for all actuators $y_{a}\left(t_{0}\right)$ is the initial output vector, T gathers the time constants, $u_{a}\left(t\right)$ contains the input signals, and $u_{\min}$ and $u_{\max}$ contain the input lower and upper limits, respectively.

The actuator output vector $y_{a}\left(t\right)$ is then computed using:

(35) $$\begin{array}{c} \begin{array}{cc} y_{a}\left(t\right)\; & =\;\left[\begin{array}{c} y_{a,1}\left(t\right)\\ \vdots \\ y_{a,n}\left(t\right) \end{array}\right]\\ \;\; & =\left[\begin{array}{c} \exp\left[-T_{1}^{-1}\left(t-t_{0}\right)\right]y_{a,1}\left(t_{0}\right)+{T_{1}}^{-1}\int_{t_{0}}^{t}\exp\left[-T_{1}^{-1}\left(t-\tau \right)\right]{\bar{u}}_{a,1}\left(\tau \right)d\tau \\ \vdots \\ \exp\left[-T_{n}^{-1}\left(t-t_{0}\right)\right]y_{a,n}\left(t_{0}\right)+{T_{n}}^{-1}\int_{t_{0}}^{t}\exp\left[-T_{n}^{-1}\left(t-\tau \right)\right]{\bar{u}}_{a,n}\left(\tau \right)d\tau \end{array}\right]\;. \end{array} \end{array}$$

**Figure 8:**
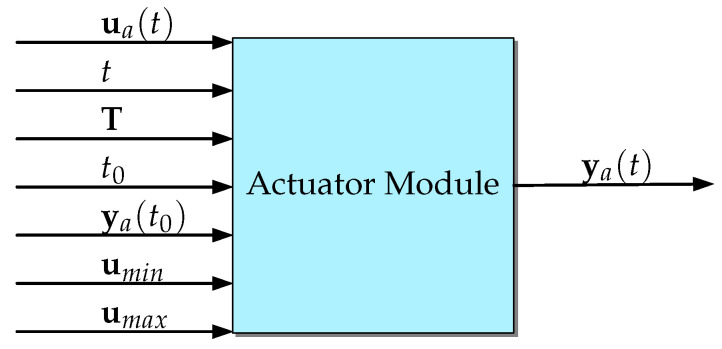
The Actuator Module as a block. Observe this Module also outputs the state of the actuator before it passes through the saturation.

**Figure 9:**
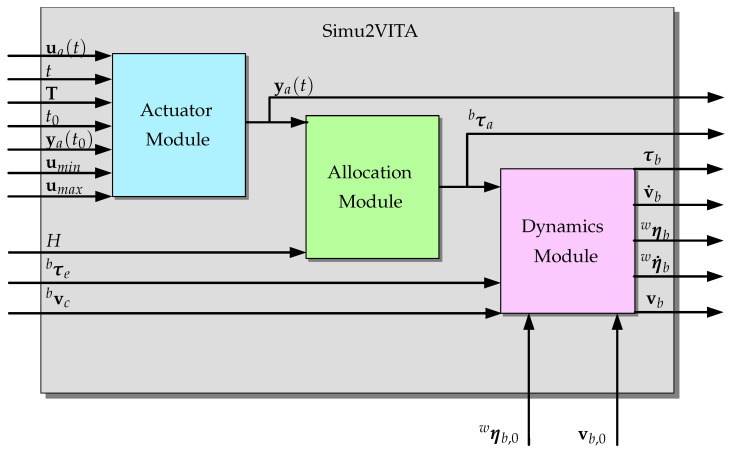
Logic connection of all three modules and their input and output signals.

Figure 8 represents graphically the Actuator Module as a block. Figure 9 shows the connection of all modules as a whole greater block, Simu2VITA. This can serve as an initial point to visualize possible ways to adapt it to other types of marine crafts other than underwater vehicles.

## 4. Experiments

**Figure 10:**
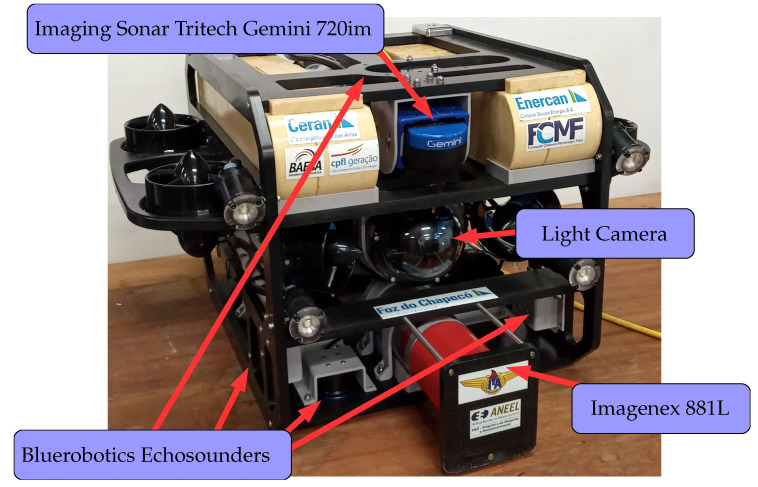
The vehicle VITA1 and its sensors.

In this section, three experiments are presented to demonstrate the flexible use of Simu2VITA: two simulated and one in a real UUV. The simulated results are then qualitatively compared with telemetry data captured when a real UUV was deployed in loco. We simulate a UUV named VITA1 [21], shown in Figure 10, which is a modified version of the BlueROV2 sold by Blue Robotics [22]. The configuration parameters that define the VITA1 dynamics were taken from a similar vehicle described in [23], and in Appendix A, it is explained how these parameters were loaded in the simulator.

VITA1 has eight fixed propellers acting as actuators and the following sensors:

- A set of four echosounders from Bluerobotics pointing outwards from the vehicle [24],
- An imaging sonar, model Tritech Gemini 720im [25],
- A profiling sonar, model Imagenex 881L [26], and
- A high-definition (1080p, 30fps) wide-angle low-light camera [27] equipped with four small lights.

**Figure 11:**
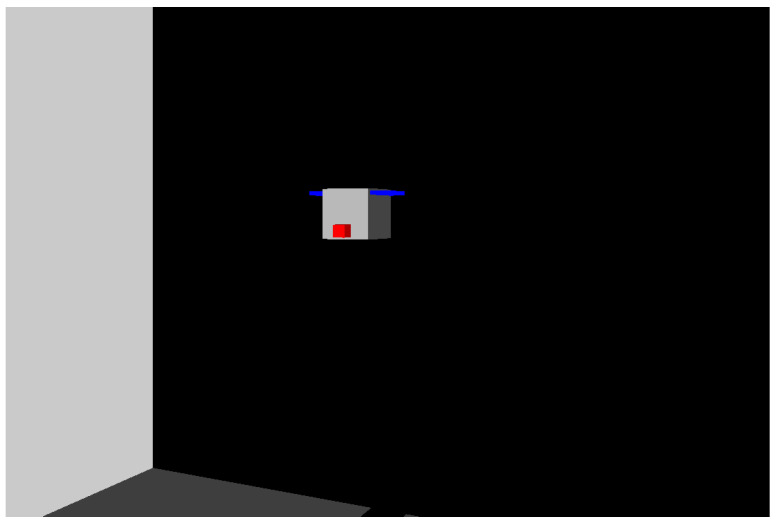
Visualization of the 3D model of the vehicle and the scenario. The red dot in front of the simulated vehicle is an allusion to the red of the Imagenex Profiling Sonar 881L. The blue lines on both sides are the representation of the “wings” carrying the propellers on VITA1. The side wall and floor of the simulated tunnel can be seen in gray and dark gray, respectively, on the left.

We use the Simulink 3D Animation toolbox for visualizing the dynamics of the vehicle. This visualization shows the vehicle pose over time as a 3D animation; see Figure 11. The echosounders are simulated as lines going out from them. The distance between an echosounder and an object is obtained when its line intersects the object. This intersection detection is made automatically by the Simulink 3D Animation toolbox.

### 4.1. Simulated Experiments

**Figure 12:**
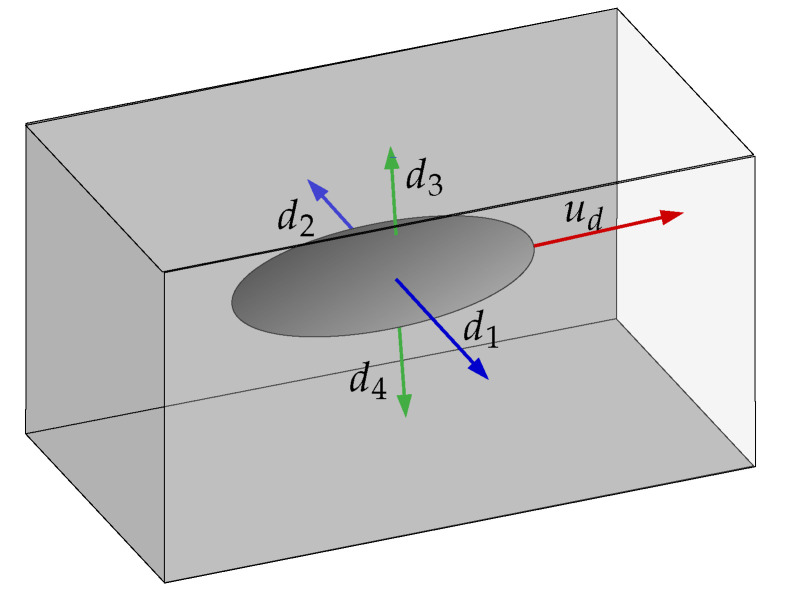
Distances measured by the VITA1 echosounders.

In the simulated experiments presented in this section, the vehicle navigates inside a fully flooded underwater straight tunnel. Two scenarios are presented each with a different path. In the first scenario, the vehicle should move with a constant desired forward speed, in the center of the cross-section of the tunnel and oriented as the tunnel main axis. The tunnel itself is oriented in the same direction as the n of the frame {w}. In the second scenario, the vehicle follows a sinusoidal vertical path, while the other objetives are are the same. To achieve these goals, two additional systems were attached to Simu2VITA: a Guidance System and a Control System. The Guidance System continuously updates the desired path the vehicle should follow. The Control System generates the command signals for the vehicle actuators such that it follows the desired path generated by the Guidance System as close as possible. The general picture of the problem can be seen in Figure 12, with the four echosounders readings ($d_{1}$ to $d_{4}$) shown as blue and green arrows and the red arrow pointing forward indicating the direction of the desired forward speed.

The Guidance System receives the desired values for the vehicle forward speed $u_{d}$, the desired vehicle orientation qwb,d, the desired offsets between lateral echosounder readings ebsw,d and vertical echosounder readings ebhe,d. The lateral and vertical distances of the vehicle to the center of the tunnel cross-section are computed using ebsw=d2−d1 and ebhe=d3−d4. These desired values are then smoothly interpolated with the current state of the vehicle and sensor readings generating a smooth path to be followed. The signal outputs of the Guidance System are used as reference values when entering the Control System. Note that these references are smooth paths meaning that for the case of speed, there is an acceleration reference, too, and for the case of offsets and orientation that are constraints on speed and acceleration.

The Control System is responsible for generating command signals to the vehicle actuators to move the vehicle. The Control System is composed of four distinct controllers: a forward speed controller, a centralization controller, an orientation controller and a stabilizer controller.

To reach the reference velocity $u_{ref}$ coming from the Guidance System, the forward speed is implemented as a PI controller and a feedforward reference acceleration term ${\dot{u}}_{ref}$ is used. The idea is that once the error between the measured forward speed of the vehicle and the reference speed approaches zero, only the reference acceleration input remains. For a constant desired forward speed, the final reference acceleration value will be zero.

The centralization controller is responsible for positioning the vehicle in center of the tunnel cross-section. It is implemented using two separated PID controllers for both lateral and vertical position correction.

The orientation controller is a non-linear controller that uses quaternion directly based on the work of Fresk and Nikolakopoulos [28].

Finally, the stabilizer controller compensates the non-linear parts of the model using a state feedback linearization approach and decoupling the motion of the vehicle. More details about the derivation and implementation of the Guidance and Control Systems are given by de Cerqueira Gava et al. [29].

The forward speed and the centralization controllers generate force commands. The orientation controller generates torque commands. To transform forces and torques into actuator inputs (propellers in this case), the simplest form was used. From Equation (21), we use the pseudo-inverse of *H* to obtain the actuators input

**Figure 13:**
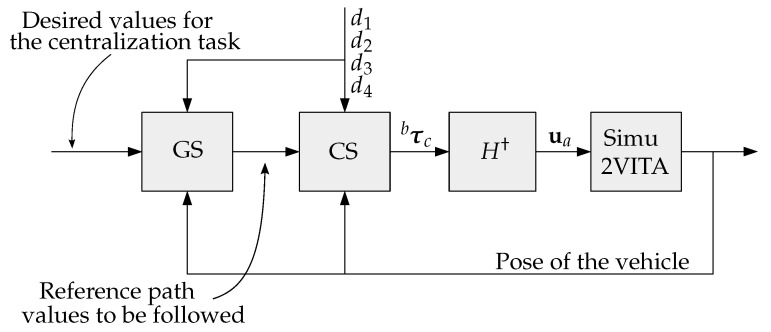
Diagram of the connection of the Guidance System (GS), Control System (CS) and Simu2VITA.

(36) ua=HT(HHT)−1︸H†τbc

with τbc being the output of the Control System of forces and torques. Figure 13 shows how the Guidance and Control Systems are connected to Sim2VITA and their respective input and output signals.

The simulation was performed using the ODE solver ode45 with a step size of 0.0025 s in MATLAB R2019a. The Guidance System runs at 20 Hz as well as the Control System controllers but the stabilizer controller is running at 400 Hz. We opted to put this high control rate to resemble the hardware we have in the real vehicle: a PixHawk micro-controller board [30] running the ArduSub software [31]. The PixHawk runs its internal stabilizer controller at 400 Hz.

For the the first scenario, we have set the tunnel entrance at the origin of the inertial system {w} alongside the direction of n; the simulated tunnel has a square profile with each side measuring 8 meters. The vehicle initial state, as explained in Section 3.1.2, is

**Figure 14:**
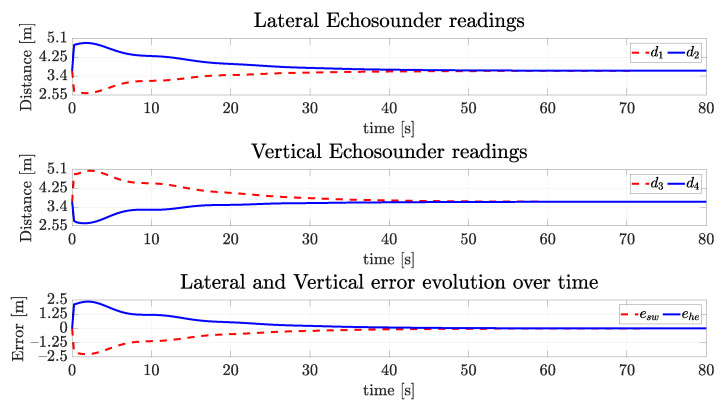
Readings of echosounders of the simulated vehicle and the vertical and horizontal errors over time for the straight path.

(37) ηwb,0=31−10.9764−0.01990.17760.1209,

(38) vwb,0=06×1,

with the quaternion part being equivalent to an orientation of $-5^{\circ }$ in roll, $20^{\circ }$ in pitch and $15^{\circ }$ in yaw. The desired final surge velocity $u_{d}$ is 0.2 m/s. The desired ebsw and ebhe are zero. The centralization task may be seen from the signals of the simulated echosounders in Figure 14. Observe the lateral and vertical echosounders readings converging to the same value (3.70 m), leading to errors ebsw and ebhe to zero. The lateral and vertical echosounders readings converge to 3.70 m, since the simulated tunnel has a square cross-section with an 8 m side length. The vehicle geometric shape in simulation is a cube with a 0.6 m side length, and the echosounders are assumed to placed at the vehicle surfaces, not at its center.

**Figure 15:**
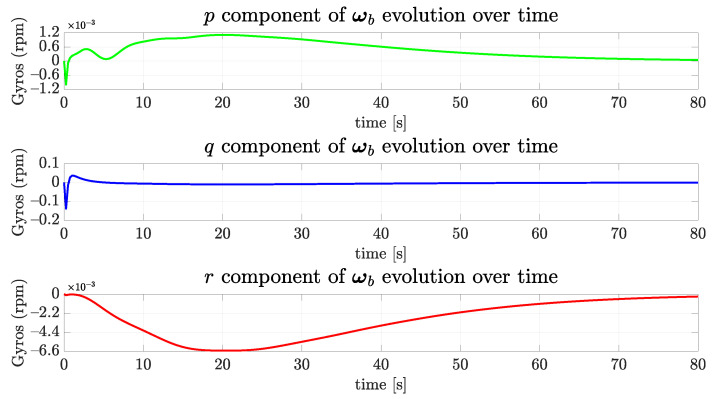
The evolution of angular speed components of the simulated vehicle over time for the straight path.

**Figure 16:**
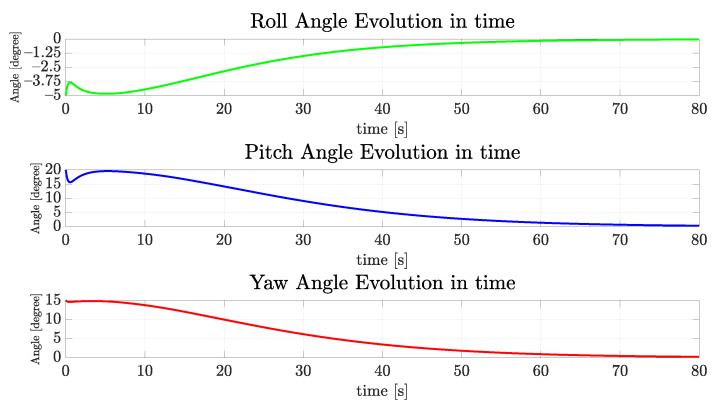
The evolution of orientation components of the simulated vehicle over time for the straight path.

Considering the regulation of vehicle orientation, the dynamics of the vehicle are stable for the roll and pitch axes, so these angles naturally converge to zero. However, the yaw angle must be actively controlled in order to follow the referencing signal. In this case, as the tunnel sagittal plane is oriented orthogonal to the coronal plane of the world frame {w} (ed-plane) and the vehicle must cruise the tunnel with nwb parallel to the walls, the desired final yaw value should be zero. Figure 15 and Figure 16 show the evolution of the angular velocities in gyros and orientation angles. They are less than $1^{\circ }$ at 80 s and slowly converge to zero.

**Figure 17:**
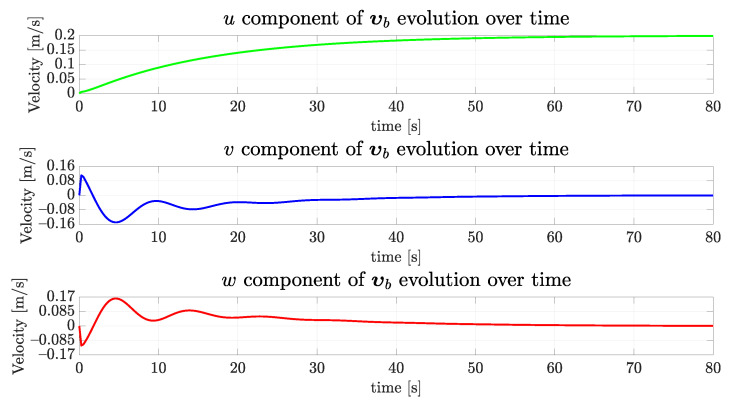
The evolution of the components of the simulated vehicle linear velocity over time for the straight path.

As the vehicle started, the simulation displaced by a meter up and to the right, and rotated, is expected to exert considerable horizontal and vertical velocities. Figure 17 shows the simulated vehicle velocity vector evolution in the three axes, as depicted in Figure 2. Observe how the desired forward velocity $u_{d}$ = 0.2 m/s is achieved, while the vehicle centralizes itself. In this case, *v* and *w* velocities evolution present a skew similar profile.

**Figure 18:**
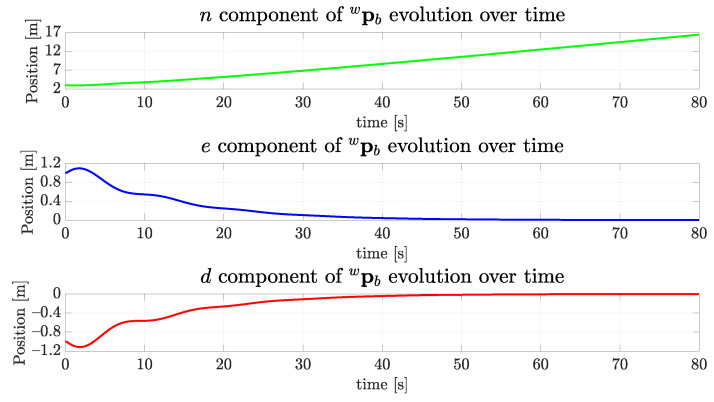
The evolution of the position components of the simulated vehicle over time for the straight path.

The evolution of components of the position pwb of the vehicle can be seen in Figure 18. As expected, the *n* component has grown as the time passed, and both *e* and *d* components converged to zero, as was previously shown in Figure 14. This happens because the center of the tunnel profile occurs at the origin of the coronal plane.

**Figure 19:**
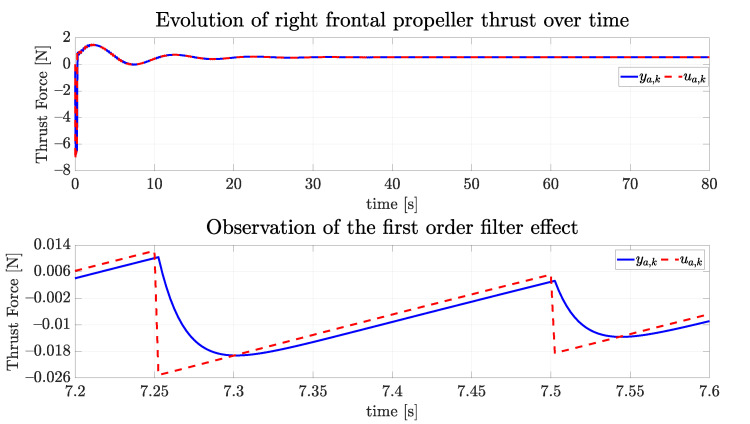
The evolution of one of the propellers over time and the effect of the first order system as the response of the actuator.

The simulated propellers were eight, all having the same lower and superior limits of 39.91 N and 51.48 N, respectively. These values are informed by the manufacturer of the real propeller [32] used in the real vehicle for the specified tension of 16 V. For the time constant we have used 0.1754 s, which is a value also used by Manhães et al. [11]. Figure 19 shows the evolution of a propeller over time, with the lower graph depicting the transitory response for a series of changing values of input.

In the second scenario, we present a sinusoidal path in heave. This path is of interest for situations when we need to collect point cloud data using the imaging sonar. The initial state is the same as in the previous case, but as the path changes, the response also changes. In this particular case, we are interested in how the vertical movement interferes with the capacity of the vehicle to keep heading toward the end of the tunnel in a constant frontal speed. The sinusoidal path is determined by a sinusoidal function with unitary amplitude and period of 17.5 s, leading to a frequency of 0.359 rad/s. The restrictions on the vertical velocity and acceleration are imposed using first and second derivatives of the sinusoidal function.

**Figure 20:**
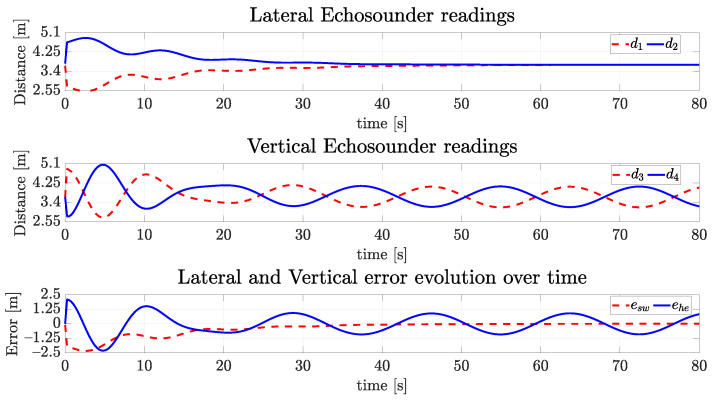
Readings of echosounders of the simulated vehicle and the vertical and horizontal errors over time for the sinusoidal path.

**Figure 21:**
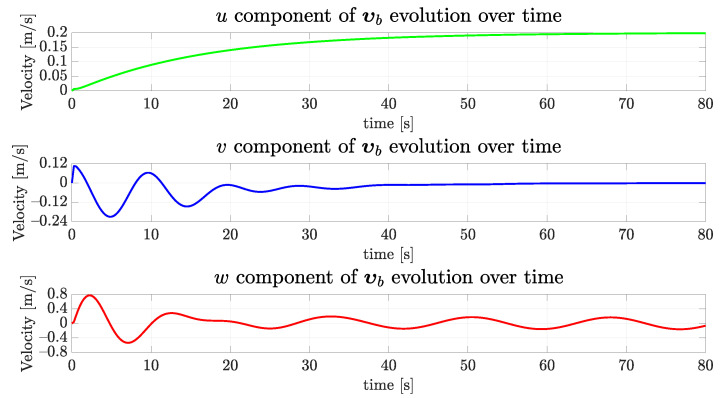
The evolution of the components of the simulated vehicle linear velocity over time for the sinusoidal path.

**Figure 22:**
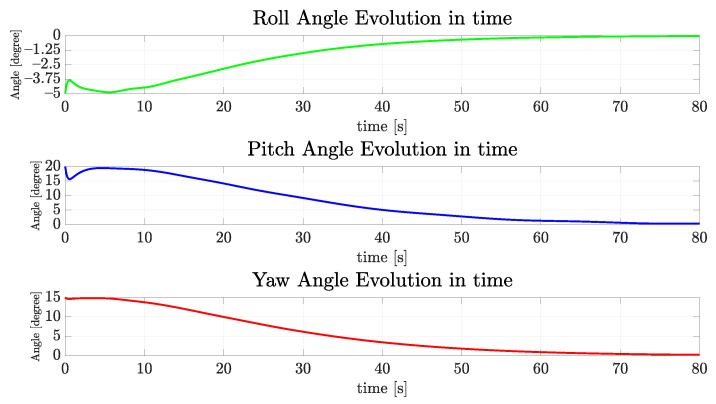
The evolution of orientation components of the simulated vehicle over time for the sinusoidal path.

**Figure 23:**
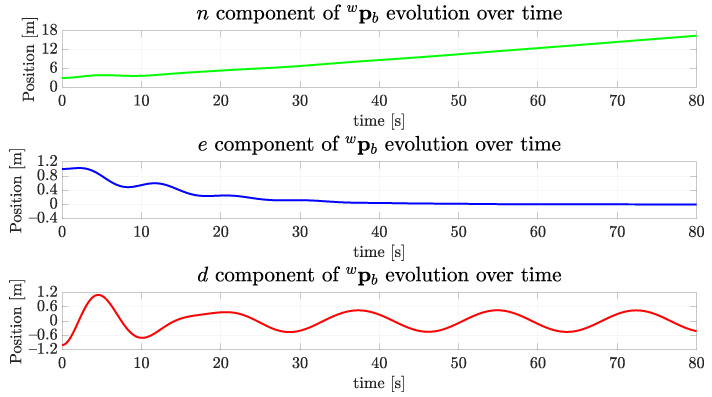
The evolution of the position components of the simulated vehicle over time for the sinusoidal path.

Figure 20 shows the evolution of the readings of the vertical and lateral echosounders. Observe the sway offset converging to zero and the heave offset stabilizing between 0.89 m and −0.91 m. In Figure 21, the forward speed converges similarly to the first simulated scenario, the lateral speed goes to zero and the vertical speed achieves an oscillatory regime as expected. The orientation can be seen in Figure 22, and the convergence to zero in all three dimensions is evident. Finally, Figure 23 shows that the desired behavior is achieved with the vehicle going forward, heading toward the tunnel with constant oscillatory vertical displacement.

### 4.2. Real Experiment

For the real experiment, the VITA1 vehicle was placed inside a hydro-power plant adduction tunnel, which is 100 m long and 3.80 m wide. The vehicle was attached to a topside station through a tether cable, with the Guidance and Control Systems executing at the station. The only controller executing embedded of the vehicle was the stabilizer controller running in the PixHawk board. The control rate of the systems previously mentioned is the same as those in the simulated experiment. For a detailed explanation of the functioning of VITA1, please refer to the work of Jorge et al. [33].

This experiment resembles the second simulated scenario. The vehicle vertical desired path ebhe is sinusoidal. This happened because there was a requirement to acquire more information with the imaging sonar. The sinusoidal path allowed us to obtain readings from the upper and lower part of the tunnel. There are still the following differences in relation to the second simulated experiment:

- The controller compensating non-linear terms of the vehicle dynamics is a cascade PID running at 400 Hz on the micro-controller PixHawk [30] using readings from its own internal accelerometers and gyrometers;
- The orientation controllers operate separately in each angular degree of freedom also using a cascade PID inside PixHawk, while the simulated vehicle used a composed orientation controller in quaternion form.

These differences happened due to the poor documentation of ArduSub, which was the firmware running inside PixHawk. Although the documentation explains the overall structure of the controller implemented in ArduSub to decouple the axis of the vehicle, the exact implementation of this controller is not explained. The opposite also happens, the customization of the stabilizing controller and orientation controller inside the ArduSub firmware is poorly documented, and understanding the source code would be difficult and costly in terms of working time. This led us to use different stabilizing controllers, but we made sure both would be sufficient to decouple the motion of the vehicle under low speed. With such difference in the controllers, we expect similar but not totally equal behavior. We expected to see the sinusoidal path in evidence, and this happened as shown in the following section.

The forward speed and the centralization controller remains with the same structure, but now, they generate inputs for the PixHawk internal controllers. The state observation algorithm used the one presented by Pittelkau [34] and embedded in the PixHawk. The vehicle departs from the entrance of the tunnel almost pointing to the desired yaw orientation of $-137^{\circ }$ and almost centralized.

**Figure 24:**
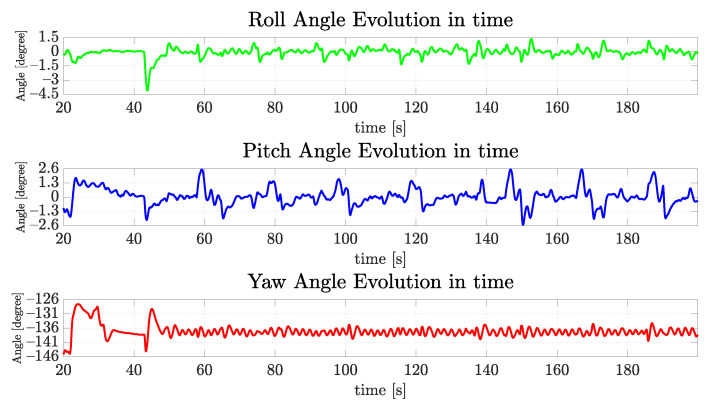
The evolution of orientation components of VITA1 over time for the real vehicle.

Figure 24 exhibits the evolution of the orientation over time, where roll and pitch remain in a well-bounded box around zero, also the yaw track and the desired yaw angle remains around it.

**Figure 25:**
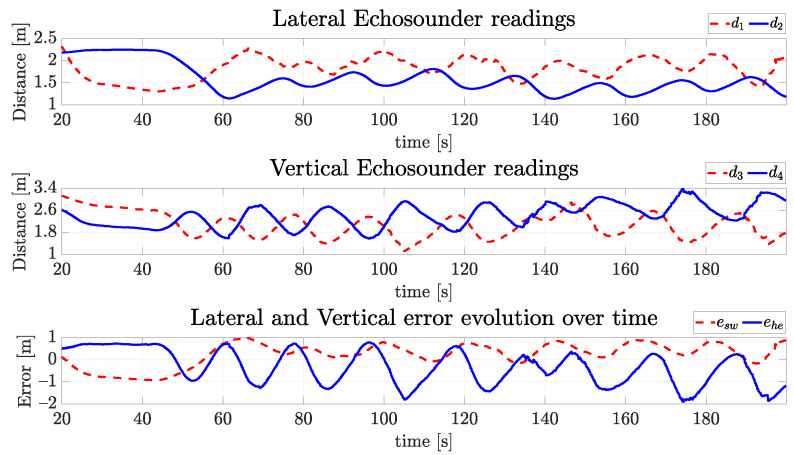
Readings of echosounders of VITA1 and the vertical and horizontal errors over time for the real vehicle.

The echosounder signals in Figure 25 show that in the horizontal movement, bouncing converges to an oscillatory pattern around 0.5 m, and the vertical sinusoidal pattern is quite evident. While the vehicle goes up and down in vertical movement, the tunnel diameter may change some centimeters due to indentations in the tunnel wall, thus contributing to this also oscillatory behavior in sway, which was not present in the simulation results.

**Figure 26:**
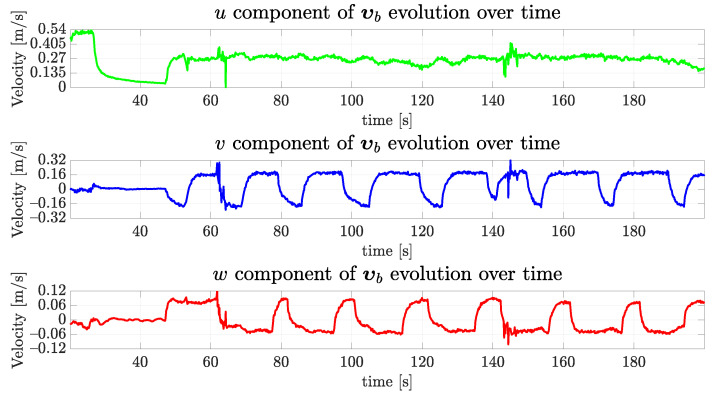
The evolution of linear velocities components of VITA1 over time for the real vehicle.

Last, observe in Figure 26 that the forward velocity is around 0.27 m/s, which is 0.02 m/s above the desired forward velocity at 0.25 m/s for the real experiment. As expected from Figure 25, there was expressive speed in the vertical direction of the vehicle. The velocities were measured using the DVL sensor A50 from Waterlinked [35] attached to the bottom of VITA1 pointing downwards.

From the previous experiments, we have shown that it is possible to use Simu2VITA to model and test controllers and behaviors for some desired vehicle, even for the case in which the simulated experimented used perfect sensing, while the real case used an Extended Kalman Filter to estimate its states. In addition, the assumption of slow decoupled movements held, as a high-rate PID was able to “linearize” the dynamics of the vehicle.

## 5. Conclusions and Future Works

This article reports the development and some use cases of Simu2VITA, which is a simulator designed for UUV simulation. The advantages of our solution over other simulators previously presented are:

- Our simulator uses an added matrix to enhance the simulation accuracy;
- Our user interface takes away the laborious work of searching through a myriad of files to enter the simulation information and concentrates these parameters to the user in a simple menu. No need for text editing.
- Inherits the easy prototyping aspect of Simulink^®^ and makes controller design quick by using visual tools.
- No joint configuration is needed.

Simu2VITA is easy to set up and facilitates the rapid prototyping and validation of concepts. Our simulator has been demonstrated to be a simple and resourceful tool when designing controllers and autonomous behaviors for a UUV. The simplicity of Simu2VITA and its easiness of use allowed our research project team to become familiar with the behavior of the real underwater vehicle before any in loco experiment.

As future work, we plan to implement simulated versions of some types of sonar sensors. There are already some models for a complex sensor such as the sonar as the one proposed by Mai et al. [36]. Another possible future work is to provide an animation model for the 3D animation system of Simulink^®^. Previous prepared controllers and behavior algorithms are in the sight of this research, too, to enable the rapid prototyping of autonomous underwater vehicles. In comparison to other simulators, Simu2VITA still lacks collision detection, but this can be implemented outside of the simulator and is a possible future improvement to be made. Another future addition to Simu2VITA is to add to it more complex dynamic models for the actuators.

## Figures and Tables

**Figure A1:**
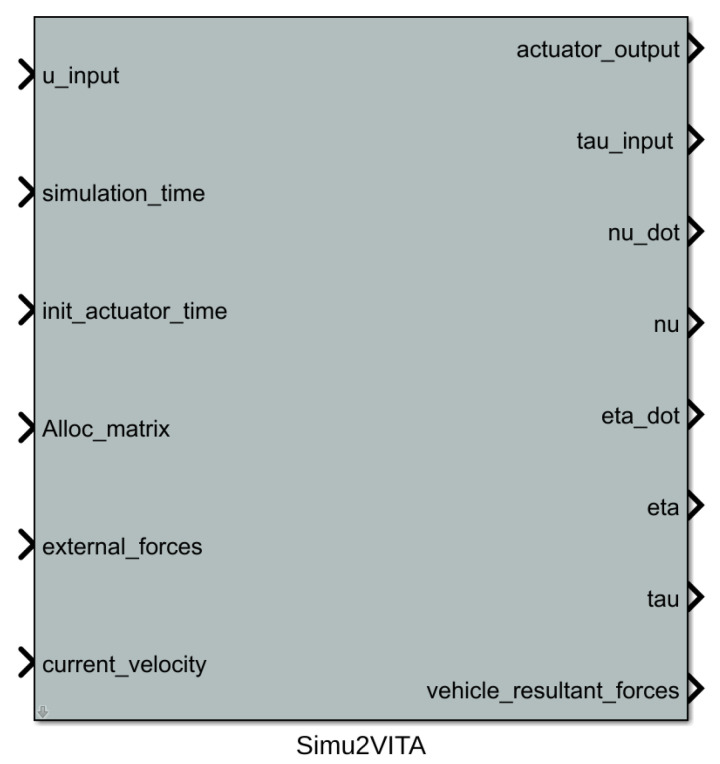
This is how the Simu2VITA block is presented in Simulink.

**Figure A2:**
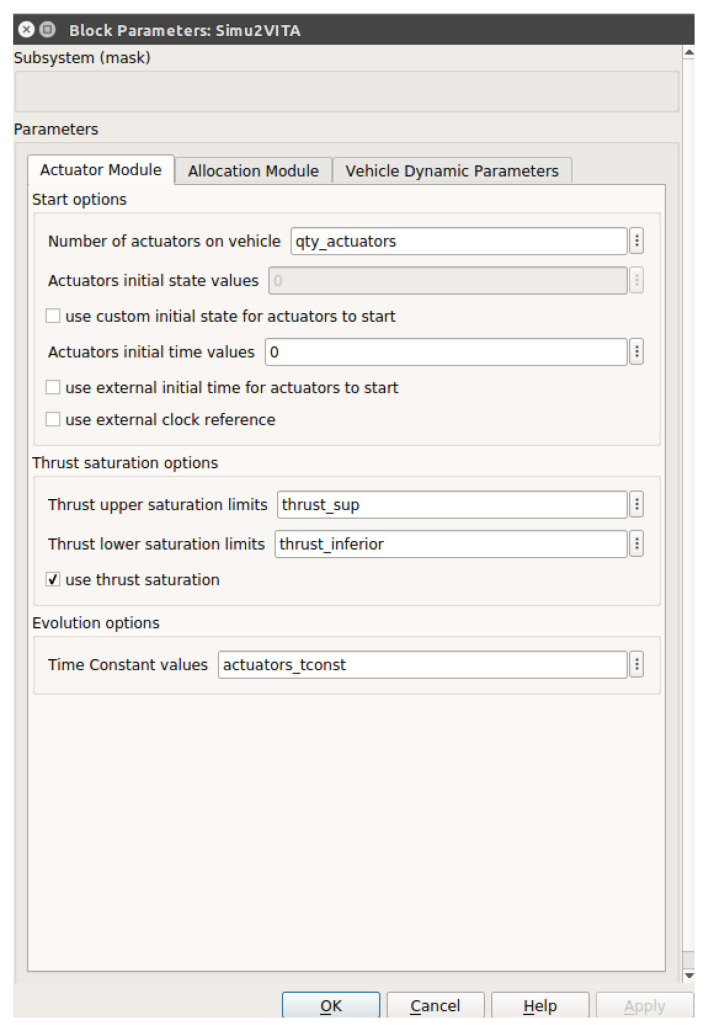
Simu2VITA interface for entering with simulation parameters for the Actuator Module.

**Figure A3:**
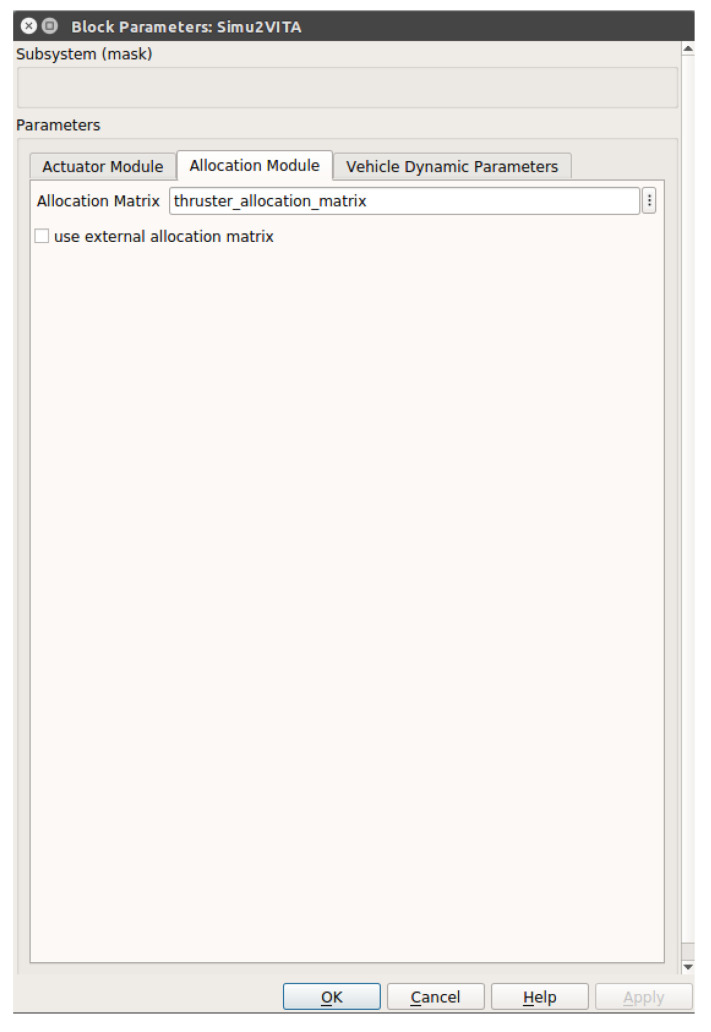
Simu2VITA interface for entering with simulation parameters for the Allocation Module.

**Figure A4:**
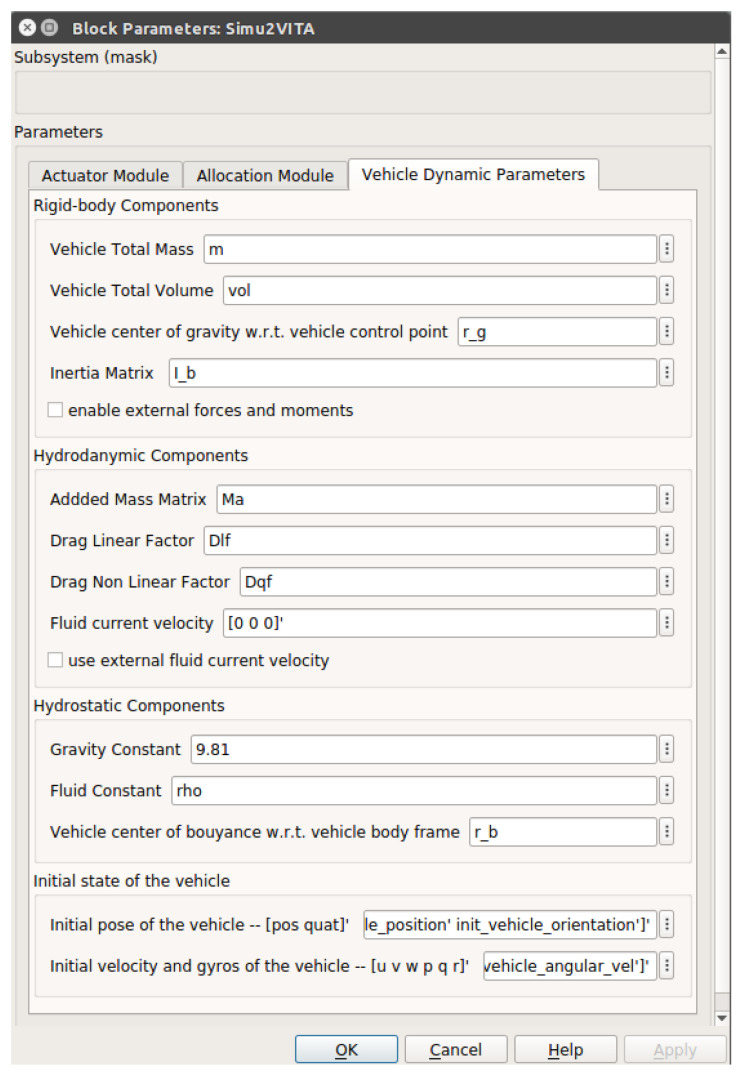
Simu2VITA interface for entering with simulation parameters for the Dynamics Module.

## Data Availability

Data is contained within the article.

## Appendix A. Simu2VITA Block on SIMULINK

Simu2VITA is a software built on top of the Matlab/Simulink^®^ framework. It is a self-contained Simulink block. Figure A1 shows the block as shown on Simulink with its inputs and outputs. Almost all inputs and outputs in Figure A1 have a correspondence to some previously mentioned variable in Section 3, except

- Input *init_actuator_time*,
- Input *simulation_time*, and
- Output *vehicle_resultant_forces*.

Starting with *init_actuator_time*, it receives a column vector n×1 containing the time an actuator will start receiving inputs, while the *k*-th element references the *k*-th actuator time to start. The *simulation_time* input enables an external clock to be used; for example, if one would like to control the vehicle in Simu2VITA using the ROS [37] network and its clock, in this case, the simulator makes the first value received as the base time and the simulation internal time is in reference to that base time. The output *vehicle_resultant_forces* is equivalent to the right hand-side of Equation (19) multiplied on the left by $\left(M+M_{a}\right)$. The other inputs are:

- *u_input* that is equivalent to $u_{a}$ in Equation (31);
- *Alloc_matrix* is equivalent to *H* matrix in Equation (26);
- *external_forces* is τbe in Equation (18); and
- *current_velocity* is vbc in Equation (9).

Outputs are:

- *actuator_output* being $y_{a}$ as in Equation (35);
- *tau_input* being τba as in Equation (18);
- *nu_dot* being v˙b as in Equation (19);
- *nu* being vb as in Equation (5);
- *eta_dot* being η˙wb as in Equation (6);
- *eta* being ηwb as in Equation (4);
- *tau* being τb in Equation (19);
- *current_velocity* is vbc in Equation (9).

Each one of the three modules of Simu2VITA has a tab dedicated to entering information. The tab for the Actuator Module needs the total number of actuators to be simulated, their initial state, the initial time they start to receive input, and if Simu2VITA is going to use some external clock base. Next, it presents the saturation options, the lower and upper limits, and one can also disable the saturation. Lastly, the time constant for each actuator is informed. See Figure A2.

The tab for the Allocation Module contains only the field for entering with a static allocation matrix, but an option to use an external source is also available. The internal machinery of Simu2VITA will correctly pick the chosen matrix based on the option of using an external allocation matrix. See Figure A3.

Information regarding the Dynamics Module involves scalars, matrices, and vectors presented on Section 3.1.1 and Section 3.1.2. From the vehicle are required its mass, volume, center of gravity and its inertia matrix. For the hydrodynamics parameters, the added mass, linear and non-linear damping factors, and the water current velocity are also needed. Observe that the damping factors are considered diagonal matrices; thus, the input is a column vector for both fields. The hydrostatic parameters are the gravity constant, the water constant and center of bouyancy of the vehicle. The last two parameters are the initial pose and the initial velocity of the vehicle. See Figure A4.
